# Gene silencing of *Sugar-dependent 1* (*JcSDP1*), encoding a patatin-domain triacylglycerol lipase, enhances seed oil accumulation in *Jatropha curcas*

**DOI:** 10.1186/1754-6834-7-36

**Published:** 2014-03-08

**Authors:** Mi Jung Kim, Seong Wook Yang, Hui-Zhu Mao, Sivaramakrishnan P Veena, Jun-Lin Yin, Nam-Hai Chua

**Affiliations:** 1 Host-Pathogen Interaction Group, Temasek Life Sciences Laboratory, 1 Research Link, National University of Singapore, Singapore 117604, Singapore; 2 Present address: Department of Plant and Environmental Sciences, Faculty of Science, University of Copenhagen, Thorvandsendsvej 40, 1871 Frederiksberg C, Copenhagen, Denmark; 3 Laboratory of Plant Molecular Biology, The Rockefeller University, New York, NY 10065, USA

**Keywords:** Inducible maker-free *JcSDP1-*RNAi, *Jatropha*, *sdp1-5*, Triacylglycerols (TAGs)

## Abstract

**Background:**

Triacylglycerols (TAGs) are the most abundant form of storage oil in plants. They consist of three fatty acid chains (usually C16 or C18) covalently linked to glycerol. SDP1 is a specific lipase for the first step of TAG catabolism in Arabidopsis seeds. Arabidopsis mutants deficient in *SDP1* accumulate high levels of oils, probably due to blockage in TAG degradation. We applied this knowledge from the model plant, *Arabidopsis thaliana*, to engineer increased seed oil content in the biodiesel plant *Jatropha curcas* using RNA interference (RNAi) technology.

**Results:**

As Jatropha is a biodiesel crop, any significant increase in its seed oil content would be an important agronomic trait. Using *A. thaliana* as a model plant, we found that a deficiency of *SDP1* led to higher TAG accumulation and a larger number of oil bodies in seeds compared with wild type (Columbia-0; Col-0). We cloned Jatropha *JcSDP1*, and verified its function by complementation of the Arabidopsis *sdp1-5* mutant. Taking advantage of the observation with Arabidopsis, we used RNAi technology to generate *JcSDP1* deficiency in transgenic Jatropha. We found that Jatropha *JcSDP1-*RNAi plants accumulated 13 to 30% higher total seed storage lipid, along with a 7% compensatory decrease in protein content, compared with control (CK; 35S:*GFP*) plants. Free fatty acid (FFA) content in seeds was reduced from 27% in control plants to 8.5% in *JcSDP1*-RNAi plants.

**Conclusion:**

Here, we showed that *SDP1* deficiency enhances seed oil accumulation in Arabidopsis. Based on this result, we generated *SDP1*-deficient transgenic Jatropha plants using by RNAi technology with a native *JcSDP1* promoter to silence endogenous *JcSDP1* expression. Seeds of Jatropha *JcSDP1-*RNAi plants accumulated up to 30% higher total lipid and had reduced FFA content compared with control (CK; 35S:*GFP*) plants. Our strategy of improving an important agronomic trait of Jatropha can be extended to other oil crops to yield higher seed oil.

## Background

The diminishing worldwide stock of fossil fuel has catalyzed a soaring demand for renewable energy sources. To meet this demand, active research has been initiated during the past several decades relating to solar, wind, tidal, and geothermal power generation. Likewise, there is also an increasing focus on biofuels, which are energy sources derived from renewable biomass. There are two main types of biofuels: bioethanol and biodiesel, which are generally used as gasoline and diesel additives, respectively. Bioethanol is mainly produced by fermentation using sugar or starch derived from crops such as sugar cane and corn, whereas biodiesel is obtained by transesterification of plant oils and animal fats. In 2010, the global biofuel production reached 105 billion liters, and provided 2.7% of the world’s energy needs for transportation. It has been forecast that biofuels may account for more than a quarter of the world’s demand for transportation fuels by 2050 [1].

Biodiesel is generally produced from oilseed crops such as oilseed rape in temperate countries and from oil palm in the tropics. In the past several years, a small tree called *Jatropha curcas*, grown in tropical and subtropical regions, has emerged as an attractive candidate crop for biodiesel production. Jatropha has several interesting attributes that make it suitable for consideration as a biodiesel plant. Its seeds contain up to 40% oil, consisting of approximately 75% unsaturated fatty acids (FAs) [2,3], with a high level (around 47%) of linoleic acid (C18:2) [4]. In addition to having high oil content and favorable oil composition for biodiesel, Jatropha plants have a short gestation period, and adapt well to a wide range of agroclimatic conditions [5,6]. Moreover, its ability to grow on marginal land reduces the possibility that Jatropha may compete with food crops for arable land.

Because Jatropha has only recently been domesticated, much work remains to be done to improve its agronomic traits either by traditional breeding or by gene technology. Given the commercial interest in Jatropha seed oil, it is not surprising that the immediate focus is on seed oil content and quality. With respect to the latter trait, Qu *et al*. [7] recently reported that gene silencing of *JcFAD2* greatly enhances the proportion of oleic acids in seeds of transgenic Jatropha. Here, we addressed the issue of increasing the levels of oil accumulation in Jatropha seeds by genetic modification.

Plant oil in seeds is stored as triacylglycerol (TAG), which consists of three FA chains (usually C16 or C18) covalently linked to glycerol. Depending on the plant source, TAGs may contain FAs of different chain lengths and degrees of saturation, and the FAs may have diverse modifications. Plant TAGs are generally stored in small organelles called oil bodies, which are assembled in the developing seeds, flower petals, pollen grains, and fruits of a huge number of plant species [8,9]. During seed germination, TAGs are hydrolyzed into FAs and glycerol, and this reaction is catalyzed by TAG lipases, which are widely distributed in plants but also found in animals and microorganisms [10]. Among the known lipases are the unorthodox patatin-like TAG lipases (PTLs), which are oil body-associated enzymes that play a major role in the initiation of TAG degradation in yeast, mammals, and insects [11,12]. During seed germination, TAG lipases initiate TAG hydrolysis into glycerol and FFAs, and the latter are metabolized through the β-oxidation pathway to release carbon sources for early seedling growth [13,14].

Recently, Eastmond [15,16] showed that the *Sugar-dependent 1* (*SDP1*) gene of *Arabidopsis thaliana* encodes a patatin-like acyl hydrolase domain. The encoded protein, SDP1, is specifically responsible for the first step of TAG degradation during Arabidopsis seed germination. This enzyme is also able to associate with the surface of the oil body and with the other reported PTLs. A T-DNA insertion *SDP1* mutant allele, *sdp1-5*, displayed growth retardation on a sugar-deficient medium, as a result of deficiency in glycerol and FFA, which are products of TAG degradation [15,16]. Eastmond [15,16] showed an accumulation of clustered oil bodies in seedling cotyledons, and higher TAG levels in *sdp1-5* than wild-type (WT) seedlings (*A. thaliana* ecotype Columbia-0 (Col-0)). Although the TAG level in dried seeds of *sdp1-5* was not reported, the findings of this Eastmond study provided an important hint about the possibility of enhancing TAG levels in Jatropha seeds by suppressing SDP1 expression. More recently, Kelly *et al*. [17] reported that silencing of *SDP1* in seeds increases seed oil accumulation in *Brassica napus*.

Here, we first investigated the effect of *SDP1* functions on lipid accumulation in Arabidopsis seeds. Next, we isolated a *SDP1* homolog from Jatropha plants, and identified the function of the *Jatropha curcas sugar-dependent 1* (*JcSDP1*) gene by genetic complementation of the Arabidopsis *sdp1-5* mutant. Finally, we used RNA interference (RNAi) technology to suppress *JcSDP1* expression in transgenic Jatropha seeds, and found that the transgenic seeds accumulate high levels of lipids.

## Results

### TAG is accumulated in mature dried seeds of *sdp1-5*

From the ABRC stock center, we obtained an Arabidopsis mutant (*Salk_076697*) with T-DNA insertion in *SDP1* (At5g04040) locus, and this mutant was designated *sdp1-5*[15]. Using RT-PCR as a screen we obtained three homozygous lines (#24, #30, and #33) of the *sdp1-5* null allele, and one line (#33) was used for further experiments (see Additional file 1). *SDP1* transcript levels in *sdp1-5* (#33) were 20-fold lower than in WT (Col-0) during the early stages of seed development (3 to 5 days after pollination (DAP)) (see Additional file 1).

**Figure 1:**
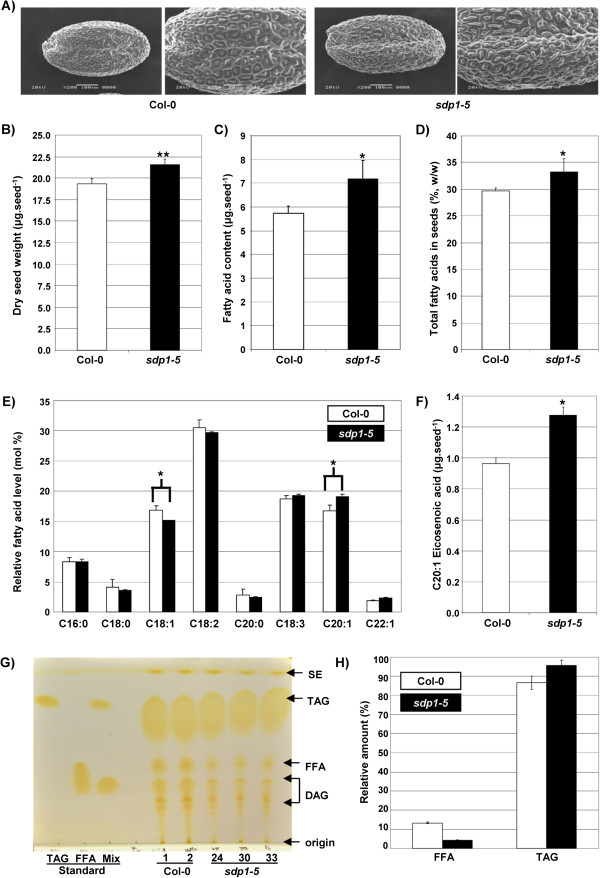
**sdp1-5 mutant accumulates higher triacylglycerol (TAG) levels than wild type (Columbia-0; Col-0) in mature seeds. (A)** Scanning electron microscopy (SEM) showing the seed surface structure of WT (Col-0) and *sdp1-5*; **(B)** comparative dry seed weight of WT (Col-0) and *sdp1-5*; **(C)** total amount of FAs per seed of WT (Col-0) and *sdp1-5*; **(D)** relative FAs of dried seeds of WT (Col-0) and *sdp1-5*; **(E)** FA profile of WT (Col-0) and *sdp1-5* seeds. **(F)** Seed eicosenoic acid (20:1) content; **(G)** thin layer chromatography (TLC) separation of neutral lipid fractions from WT (Col-0) and three lines of *sdp1-5* null mutant*;* 300 μg of neutral lipids were fractionated by TLC on silica gel plates. DAG, diacylglycerol, FA, fatty acid; FFA, free fatty acid (oleic acid), Mix, mixture of TAG and FFA; SE, sterol ester; TAG, Triacylglycerol (triolein). **(H)** Profiling of relative amounts of FFA and TAG by GC/MS. The absolute amount was calculated using C15:0 as an internal control by comparing their peak areas. *P?<?0.05 or **P?<?0.01 versus WT (Col-0). Each experiment was performed with 100 seeds per line with 5 biological replicates. Error bar shows standard deviation (SD) (n?=?5). DW, dry weight.

We first examined the effect of *SDP1* deficiency on seed development, total FA content, and FA profile. Using scanning electron microscopy (SEM), we found that *sdp1-5* seeds were slightly larger than those of WT (Col-0) in length and width (Figure 1A; see Additional file 2). In addition, *sdp1-5* seeds displayed an increase in dry weight of around 11.5% compared with WT. To investigate the role of *SDP1* in lipid accumulation in seeds, total FA content and FA composition in dried seeds were compared between WT (Col-0) and *sdp1-5* mutant. The average dry seed weight of WT (Col-0) was about 19 μg, containing approximately 5.54 μg of total FAs (Figure 1B, C). The seed lipid content obtained for the WT was very similar to those reported by others, which is around 30 to 35% of dry seed weight. However, *sdp1-5* seeds had an average dry weight of around 22 μg per seed, containing 7.17 μg of total FAs. Therefore, the levels of FAs in *sdp1-5* seeds were about 10% higher than found in WT (Col-0) seeds (Figure 1D). In addition, in *sdp1-5* seeds, there was a clear increase in the relative proportion of unsaturated FAs, such as eicosenoic acid (C20:1) (Figure 1E, F). To characterize TAG accumulation in mature dried seeds, we analyzed total neutral lipid from WT (Col-0) and three homozygous lines of *sdp1-5* null allele by thin layer chromatography (TLC) on silica gel plates, and found reduced levels of FFAs in *sdp1-5* compared with WT (Col-0) (Figure 1G). Triolein and oleic acid were used as standards of TAG and FFA, respectively. To obtain quantitative data, we analyzed the samples by gas chromatography and mass spectrometry (GC/MS), using pentadecanoic acid (C15:0) as an internal control for quantification. The *sdp1-5* mutant had about 4.25% FFA and 95.75% TAG, compared with 13.35% FFA and 86.65% TAG in WT (Col-0) (Figure 1H).

To examine the molecular basis of these changes in lipid content and FA profile, we analyzed transcript levels of key FA biosynthetic genes. We found an increased accumulation of eicosenoic acid, a very long chain FA that has been used as a metabolic marker for storage TAGs formation in Arabidopsis seeds [18]. *sdp1-5* seeds accumulated 1.3 μg of eicosenoic acid per seed, which was 40% higher than that of WT (Col-0) seeds. The increased eicosenoic acid level in *sdp1-5* seeds was probably due to the up-regulation of the *fatty acids elongase 1* (*FAE1*) gene (see Additional file 3). Our results suggest that *SDP1*-deficiency is closely correlated with seed size augmentation, the relative proportion of unsaturated FAs, and the accumulation of TAGs in mature seeds.

### *sdp1-5* mutant has an increased number of oil bodies in dried seeds

**Figure 2:**
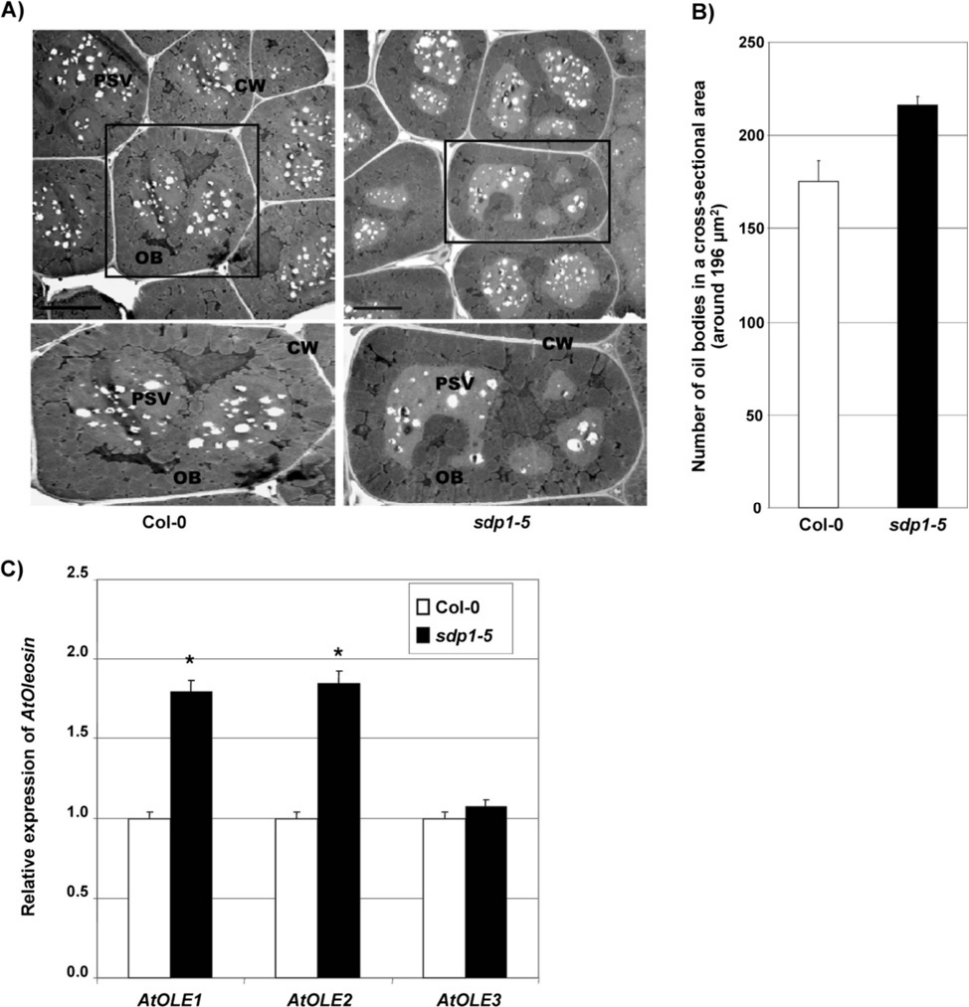
***sdp1-5*mutant seeds produce more oil bodies. (A)** Transmission electron microscopy (TEM) analysis of oil body distribution in wild type (Columbia-0; Col-0) and *sdp1-5* mature seeds. Scale bar?=?5 μm. CW, cell wall; OB, oil body; PSV, protein storage vacuole. **(B)** Number of oil bodies per cell in mature seeds of WT (Col-0) and *sdp1-5*. A cell has an average of 196 μm^2^ cross-sectional area. Values are mean?±?SD; n?=?5. **(C)** Expression levels of *AtOLE1* (At4g25140*), AtOLE2 (*At5g40420), and *AtOLE3* (At5g51210) in early developing stages of seeds (3 to 5 DAP) from WT (Col-0) and *sdp1-5. Actin1* (At2g37620) was used as an internal control. *P?<?0.05, **P?<?0.01, or ***P?<?0.001 versus WT (Col-0). There were five biological replicates. Values are mean?±?SD; n?=?5. DAP, days after pollination.

Plant storage lipids, predominantly TAGs, are sequestered by monolayer phospholipids along with embedded small proteins (such as oleosin) to form oil bodies [19]. Previous work has highlighted the importance of oleosin for oil body structure and TAG accumulation in mature seeds. We used transmission electron microscopy (TEM) to analyze the formation of oil bodies in *sdp1-5* seeds. Mutant *sdp1-5* seeds contained an increased number of oil bodies but they were relatively smaller in size compared with those in WT (Col-0) seeds (Figure 2A). The cross-sectional area of one mature seed cell was about 196 μm^2^. Within this area, there were around 175 oil bodies in WT (Col-0), but about 216 oil bodies in *sdp1-5*, representing an increase of approximately 23% (Figure 2B). Because oleosins are important proteins for oil body formation in seeds, we investigated expression levels of three different oleosin genes in *sdp1-5* by real-time quantitative PCR (qPCR). Transcript levels of *AtOLE1* (At4g25140) and *AtOLE2* (At5g40420) were two-fold higher in *sdp1-5* compared with WT (Col-0) (Figure 2C). These results suggest that the increased number of oil bodies in *sdp1-5* is possibly due to the enhanced expression of oleosins.

### *JcSDP1*, an ortholog of *AtSDP1*, rescues *SDP1* deficiency in Arabidopsis

**Figure 3:**
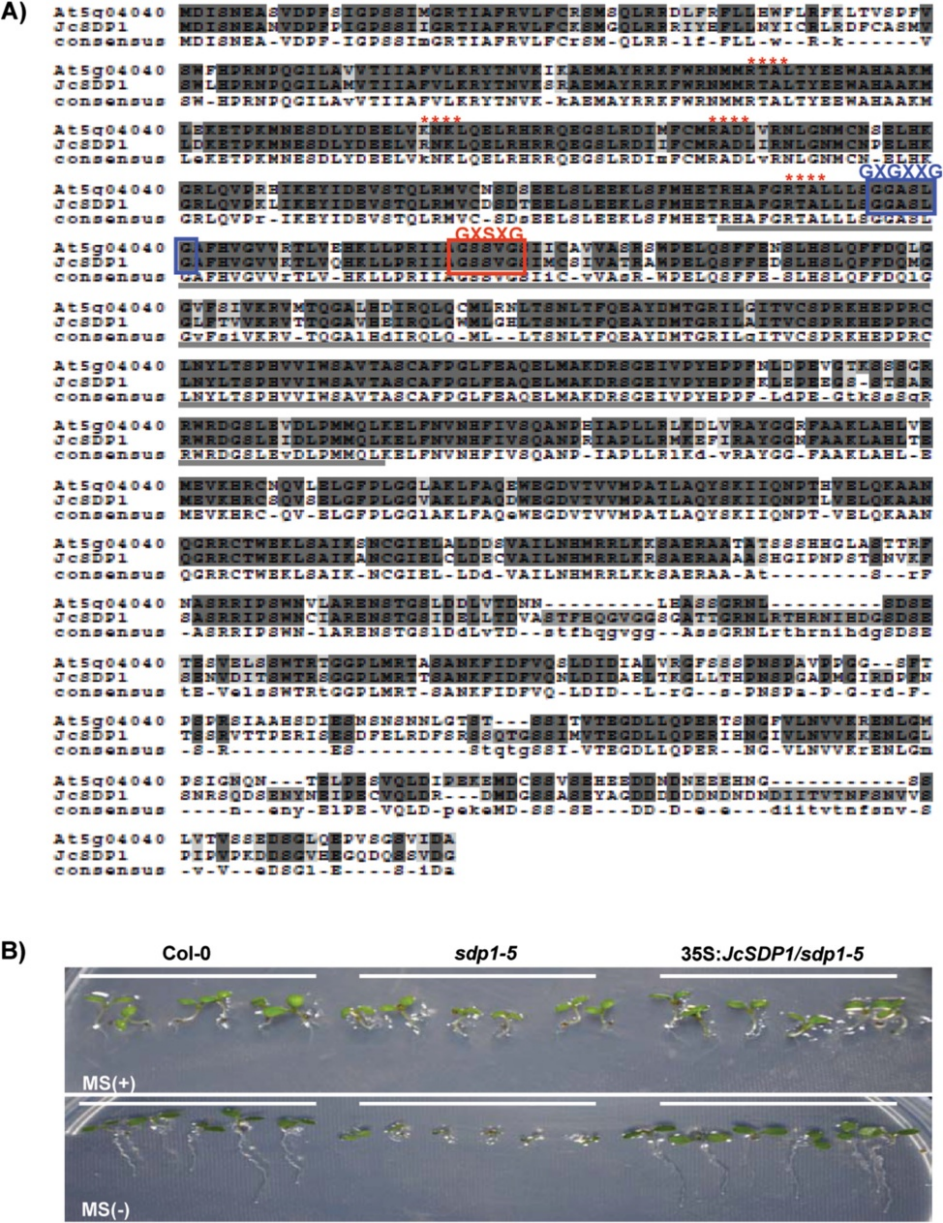
**Cloning of*JcSDP1*gene from Jatropha plants, and complementation of the Arabidopsis*sdp1-5*mutant. (A)** Deduced amino acid sequence of JcSDP1 and alignment to the AtSDP1 sequence. Bold line, patatin domain; star (*****); site-1 protease (S1P) target sequence RXXL; GXGXXG, oxyanion hole motif; GXSXG, lipase consensus motif with catalytic serine. **(B)** Complementation of *sdp1-5* with *JcSDP1*. WT (Col-0), *sdp1-5*, and 35S:*JcSDP1*/*sdp1-5* transgenic plants were grown on MS medium with or without sucrose (1%).

To investigate the impact of SDP1 deficiency in an oil seed crop, we isolated full-length *JcSDP1* cDNA from Jatropha seed RNA samples using 5′ and 3′ circularization-based rapid amplification of cDNA ends (cRACE) technology. *JcSDP1* encodes a protein of 858 amino acids with a molecular mass of approximately 96 kDa. A BLAST search revealed that JcSDP1 has high sequence homology (76%) to the Arabidopsis SDP1 (At5g04040). JcSDP1 has at least three predicted trans-membrane domains and four Site-1 protease (S1P) target sequences at the N terminus (RXXL). Moreover, JcSDP1 has a conserved patatin domain with lipase activity sequences such as the oxyanion hole motif (GXGXXG), and a lipase consensus motif with a catalytic serine (GXSXG) (Figure 3A). The high similarity of sequences and domains between JcSDP1 and AtSDP1 implied a possible similar function of JcSDP1 in TAGs metabolism. To investigate the function of JcSDP1 in TAG degradation during the early stages of seed germination, we transformed a 35S:*JcSDP1* construct into the Arabidopsis *sdp1-5* mutant. The Arabidopsis *sdp1-5* showed retarded growth on sugar-depleted MS medium, which could be rescued by sucrose (1%) supplementation [15]. We found that 7-day-old seedlings of *sdp1-5* plants expressing the heterologous *JcSDP1* displayed normal growth on MS medium without sucrose, whereas the growth of *sdp1-*5 mutant was retarded (Figure 3B). This result indicates that *JcSDP1* is able to rescue the retarded growth phenotype of *sdp1-5*, providing evidence that *JcSDP1* is an ortholog of *AtSDP1*.

### Characterization of *JcSDP1* promoter

**Figure 4:**
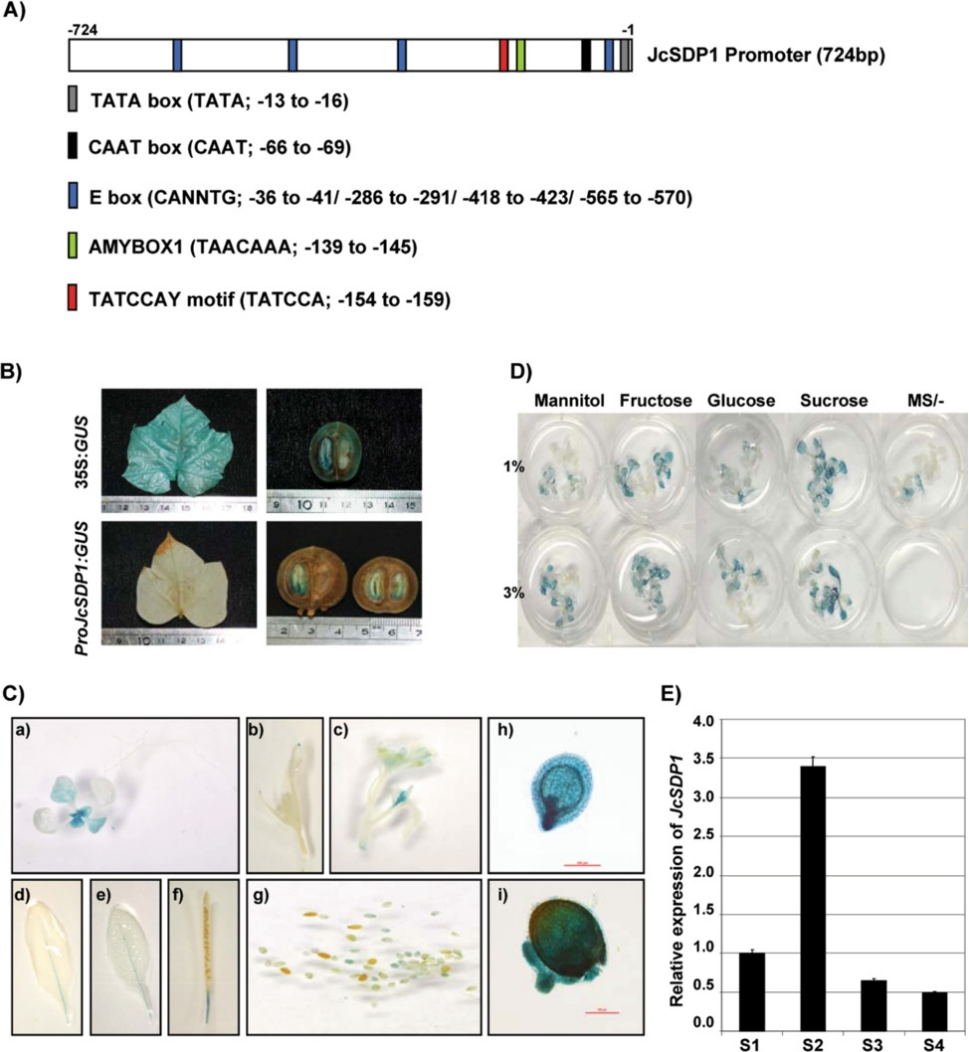
***JcSDP1*promoter and its expression analysis in*ProJcSDP1:GUS*transgenic plants. (A)** Composition of putative *cis* elements in *JcSDP1* promoter. **(B)** Transient *ProJcSDP1:GUS* expression in Jatropha fruit and leaf. **(C)** Heterologous expressions of *ProJcSDP1:GUS* in transgenic Arabidopsis plants: (a) 14-day-old seedling, (b, c) inflorescence, (d, e) rosette leaf, (f) siliques, (g) seeds at different developing stages, (h) young seeds (3 to 4 DAP, globular-stage embryo), (i) mid-stage seeds (9 to 10 DAP, mature green embryo). DAP, days after pollination. **(D)** Sugar-dependent expression of *ProJcSDP1:GUS* transgenic plants (14?=?day-old seedling). Different sugar sources (1% or 3% fructose, glucose, or sucrose) were used, and mannitol was used as a control for osmotic stress. **(E)***JcSDP1* gene expression profile using real-time quantitative PCR at different seed development stages (S1, 1 WAF; S2. 2 to 3 WAF; S3; 4 to 6 WAF; and S4; 7 to 8 WAF) *JcTubulin* expression levels were used as an internal control*.* Values are given as mean?±?SD (n?=?3). WAF, weeks after fertilization.

To specifically silence *JcSDP1* gene expression in Jatropha, we isolated a native *JcSDP1* promoter fragment from Jatropha genomic DNAs using a GenomeWalker kit. We cloned an approximately 0.7 kb fragment of *JcSDP1* proximal genomic locus to the 5′-untranslated region (UTR), which contained several putative *cis* elements for gene expression and regulation. This region of the *JcSDP1* promoter contains a TATA box and a CAAT box, located at positions −13 to −16 and −66 to −69, respectively. The promoter region also contains two putative sugar-responsive elements, TATCCA and TAACAAA, found in the α- amylase gene [20,21], located at positions −154 to −159 and −139 to −145, respectively. In addition, the promoter fragment includes four E-box motifs, CANNTG [22,23], which are likely to be involved in seed-specific expression (Figure 4A).

We examined if the *JcSDP1* promoter would show seed-specific expression in homologous and heterologous systems. To transiently analyze *JcSDP1* expression in a homologous system, we introduced the *ProJcSDP1:GUS* fusion gene into the developing fruits and leaf of Jatropha. The *β-glucuronidase* (*GUS*) fusion gene was transiently expressed in developing Jatropha fruits, especially the endosperm part of the seed, whereas leaf tissues did not show any *GUS* expression (Figure 4B). Control experiments demonstrated the expression of 35S:*GUS* in leaves as well as in seeds. These results suggest that the *JcSDP1* promoter has seed-specific expression in Jatropha plant, whereas the CaMV35S promoter shows constitutive expression. We also analyzed *JcSDP1*-GUS expression in developing seeds of transgenic Arabidopsis plants expressing the *ProJcSDP1:GUS* transgene. *GUS* expression was highly elevated at 3–4 DAP (globular embryos) (Figure 4C) and also at 9–10 DAP (mature green embryos) (Figure 4C-h and 4C-i) following the developmental stages described by Le *et al*. [24]. These expression patterns were consistent with the expression pattern of *JcSDP1*, which was highly activated at stage S2 (3 WAF) (Figure 4E). Therefore, the *JcSDP1* promoter is controlled in a seed-specific and development-dependent manner.

Using a *cis*-element prediction program, we found that the *JcSDP1* promoter carries two sugar-responsive α-amylase elements, TATCCA and TAACAAA [20,21]. To investigate the sugar responses of *JcSDP1*, we used 14-day-old transgenic Arabidopsis seedlings harboring the *ProJcSDP1:GUS* transgene. We examined the effects on *GUS* expression of sucrose, glucose, or fructose (1% or 3% w/w) supplementation in MS (−) media. As a control, mannose was used as a source of osmotic stress. Although we did not perform quantitative GUS assays, we found that the *JcSDP1* promoter at least showed sugar-dependent expression, which was especially responsive to sucrose and fructose (Figure 4D).

### *JcSDP1-*RNAi transgenic Jatropha plants accumulated increased levels of storage lipids in their endosperm

**Figure 5:**
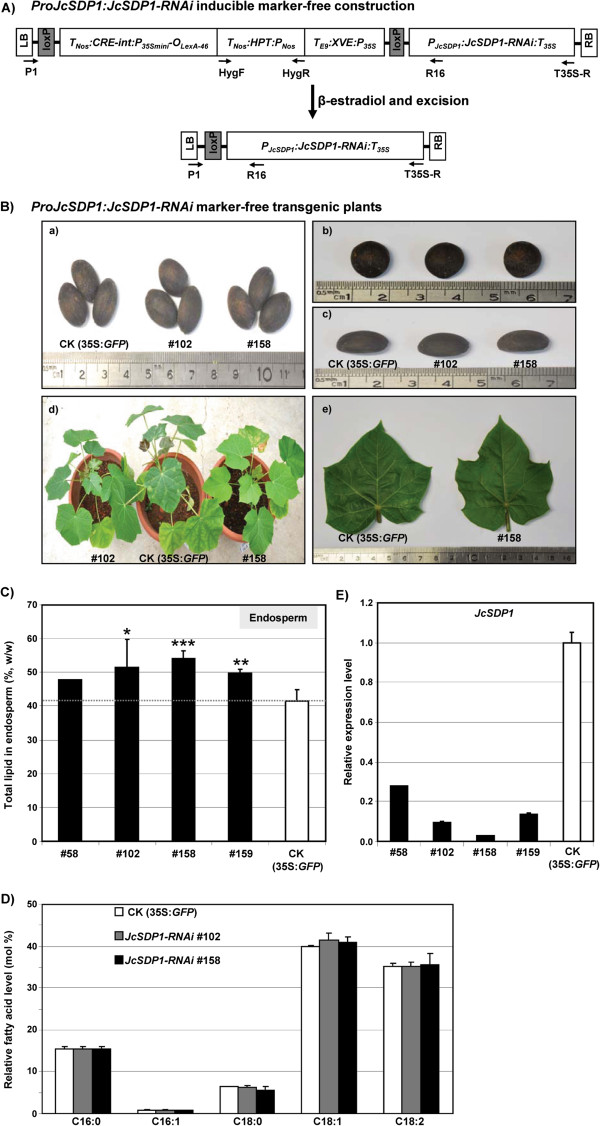
**Production of*ProJcSDP1:JcSDP1-*RNAi transgenic Jatropha plants and analysis of seed oils. (A)** Structure of the inducible *ProJcSDP1:JcSDP1-*RNAi marker-free construct. **(B)***ProJcSDP1:JcSDP1-*RNAi marker-free transgenic plants: (a-c) T1 generation of dried seeds; (d) overall phenotype of T1 transgenic plants generated from T1 embryos; (e) size comparison of the fifth leaf between control (CK; 35S:*GFP*) plants and the *JcSDP1-*RNAi transgenic line #158. **(C)** Analysis of total lipid content in mature endosperm of T1 seed. **(D)** Fatty acid profile in *JcSDP1-*RNAi transgenic lines. **(E)***JcSDP1* transcript levels in transgenic Jatropha lines. *JcTubulin* expression levels were used as an internal control*.* Numbers refer to transgenic line numbers. Asterisks indicate a statistically significant difference compared with the control, *P?<?0.05, **P?<?0.01, ***P?<?0.001 versus control (CK; 35S:*GFP*) plants, in different biological replicates.

We generated *JcSDP1-*RNAi transgenic Jatropha plants to see if a reduction of *SDP1* expression in Jatropha would also lead to increased seed oil accumulation. As we wanted to specifically control lipid accumulation in the mature seeds without ectopic effects, the *JcSDP1-*RNAi transgene was placed under the control of the native *JcSDP1* promoter, which is seed-specific. Through a two-step selection process using hygromycin and β-estradiol, marker-free or non-marker-free transgenic plants were screened. These plants were further confirmed by genotyping with specific primer sets for *HygF* and *HygR* for the hygromycin-resistance gene, and *P1* and *R16* or *P1* and *T35S-R* for the marker-free transgene. Based on the genotyping results, we obtained chimeric (heterozygote) marker-free plants in which the antibiotic selection marker was partially removed by homologous recombination. We recovered several independent lines in which growth patterns, growth rates, leaf number, and leaf size were all normal (Figure 5B–E). In contrast to the enlarged seed size found in Arabidopsis *sdp1-5*, T1 mature seeds from *JcSDP1-*RNAi plants were normal in size (Figure 5B-a to -c). To investigate total lipid content in T1 mature seeds*,* we carefully separated endosperms from embryos. The endosperms were used for further molecular and biochemical analysis, while the embryos were regenerated to maintain the transgenic lines.

After transgenic plants were fully mature, we harvested mature seeds from four individual transgenic plants for further studies. Compared with control (CK; 35S:*GFP*) plants, all tested transgenic lines accumulated increased total lipid content per dry seed weight in their endosperm (Figure 5C). Endosperm of the best transgenic line (#158) accumulated total lipid content to about 54% of the dry seed weight; this represented a 30% increase of total lipid in transgenic plants compared with control (CK; 35S:*GFP*) plants.

We analyzed *JcSDP1-*RNAi transgenic line #158 to investigate possible changes in protein and carbohydrate content along with the increased in lipid accumulation in endosperm. The protein content per endosperm of *JcSDP1-*RNAi decreased by about 7% compared with the CK (35S:*GFP*) plant (see Additional file 4). However, no difference in the carbohydrate content was detected between the two.

To characterize TAG accumulation in mature endosperm, we used TLC on silica gel plates to analyze total neutral lipid from control (CK; 35S:*GFP*) plants and from three individual lines of #158 transgenic plants. There were reduced levels of FFAs in the #158 transgenic line compared with control (CK; 35S:*GFP*) plants (see Additional file 5). In addition, the #158 transgenic plants had about 8.49% FFA and 91.50% TAG compared with 26.83% FFA and 73.17% TAG in CK (35S:*GFP*) plants (see Additional file 5). Transgenic plants of #158 also showed increased C18:1 and C20:0 in relative FA profiles of TAG (see Additional file 5). We verified the number of T-DNA insertions in the best transgenic lines by Southern blot analysis (see Additional file 6). Among the T1 progeny plants of the parental line #158, we recovered a line (#158-8) that carried a single T-DNA insert, and another line (#158-11) with multiple inserts. Regardless of the copy number of insertion, both #158-8 and #158-11 showed a very similar level of total seed lipid accumulation (see Additional file 6).

To determine possible alterations in FA profiles of *JcSDP1-*RNAi transgenic lines, we analyzed extracts by GC/MS (GCMS-QP2010, Shimadzu, Japan). Figure 5D shows that *JcSDP1-*RNAi transgenic plants displayed a very similar FA profile to that of CK (35S:*GFP*) plants.

In contrast to the changes of FAs in the Arabidopsis mutant *sdp1-5*, we were unable to detect any significant accumulation of C20:1 (<0.5% of total TAGs) in the transgenic Jatropha lines. The Arabidopsis genome encodes *FAE1*, which is involved in eicosenoic acid biosynthesis, and no homolog of this gene is present in *J. curcas*[25]. This difference explains the very low eicosenoic acid levels in Jatropha seeds. In addition, Jiang *et al*. [25] did not detect any *FAD3* transcripts in Jatropha seeds during the filling stage, an observation that is consistent with the much reduced seed 18:3 levels (see Additional file 5). Nevertheless, we observed reductions in *JcSDP1* transcript levels in *JcSDP1-*RNAi transgenic lines (Figure 5E). Moreover, the increased lipid accumulation in seeds was inversely correlated with the residual *JcSDP1* transcript levels as assayed by real-time qPCR. Taken together, these results indicate that that suppression of *JcSDP1* expression using RNAi technology can promote increased oil accumulation in dried mature Jatropha seeds.

## Discussion

### *SDP1* deficiency enhances total seed oil accumulation in Arabidopsis and Jatrohpha

The breakdown of TAGs into FAs is initiated by lipases, many of which have been identified in eukaryotes including plants, yeasts, and animals [11,12,26]. One of the characterized lipases is encoded by the Arabidopsis *Sugar-dependent 1* (*SDP1*). This lipase contains a patatin-like domain, and is tightly associated with the oil body [13,27,28]. Although three *SDP1* homologous genes, *SDP1-LIKE (SDP1L)*, *ADIPOSE TRIGLYCERIDE LIPASE-LIKE (ATGLL)*, and *COMPARATIVE GENE IDENTIFIER-58-LIKE (CGI58L)*, have been identified in Arabidopsis, only *SDP1L* plays an important role in storage lipid mobilization along with *SDP1*[29]. Besides its role in seed germination, SDP1 is also active in TAG hydrolysis during seed desiccation stage [15].

Seed development in Arabidopsis consists of two major phases: early embryogenesis, which is completed within 6 DAP, and seed maturation [24,30]. The latter process can be further defined into three sub-stages: early, mid, and late maturation. In the early maturation stage, embryos accumulate increasing amounts of starch, and begin to synthesize storage oils and proteins (7 to 10 DAP). During the mid-maturation stage (11 to 16 DAP), the amount of starch is dramatically reduced, and this is inversely related to the accumulation of oils and proteins. Finally, in the late maturation stage, which is also known as the desiccation stage, embryos gradually become metabolically quiescent, with the exception that they synthesize sugars such as sucrose and trehalose to maintain their integrity during the desiccation processes [31,32]. During the desiccation and maturation stages of *Brassica napus* seed development, at least 10% of seed storage lipid is metabolized through beta-oxidation and the glyoxylate cycle to provide carbon sources for respiration, and to a lesser extent, synthesis of amino acids that serve as precursors for the continuing synthesis of seed storage proteins [14,17]. Many enzymes, such as malate synthase, isocitrate lyase, 3-ketoacyl coenzyme A (CoA) thiolase, hydroxyacyl CoA dehydrogenase, enoyl hydratase, and phosphoenolpyruvate carboxykinase, are needed to metabolize TAG [11,12,26]. The Arabidopsis *sdp1/sdp1L* double knockout mutants display a similar phenotype as the glyoxylate cycle mutant *icl* or *icl2*, which lacks isocitrate lyase; these mutants are unable to synthesize carbohydrates from FAs [29,33]. In *sdp1-5*, TAG degradation is almost completely blocked because SDP1 is the first enzyme involved in degradation of seed storage lipid from TAG to DAG, therefore a reduction in lipid degradation is expected to reduce the amount of amino acids and hence accumulated seed storage proteins [15,29].

Based on these studies, we hypothesized that *SDP1* deficiency might block TAG metabolism during the late maturation stage, thereby reducing the loss of TAG. To test this hypothesis, we isolated the Jatropha *SDP1* homolog, and demonstrated its functional equivalence to the Arabidopsis *SDP1* by genetic complementation of the Arabidopsis *sdp1-5* mutant (Figure 3B). Moreover, we showed that *SDP1* deficiency generated by RNAi technology produced a notable increase in seed oil accumulation in transgenic Jatropha plants. This increase was accompanied by a decrease in endosperm protein content with no significant change in the carbohydrate content. In contrast to seed storage protein, reduction of *SDP1* did not have any measurable impact on seed carbohydrate content. The mechanisms underlying these differences should be addressed in future investigations.

Prior to our study, *SDP1*-RNAi lines of *B. napus* were shown to have enhanced accumulation of seed oil [17]. Extending those findings, our work here shows that the strategy of suppressing *SDP1* expression in seed can be applied to enhance seed oil accumulation in the perennial shrub Jatropha as well. It is possible that seeds of *SDP1*-deficient transgenic Jatropha plants may be partially blocked in the desiccation process, which may negatively affect their long-term shelf life. Moreover, the inhibition of TAG degradation may retard seedling growth and reduce seedling vigor in Jatropha, even though this is not the case for the oilseed rape transgenic lines [17]. However, in the case of Jatropha, these issues can be mitigated by the industrial scale production of clonal transgenic plants by tissue culture. The issue of reduced seedling vigor can also be obviated by germinating seeds in a sucrose-supplemented medium and selling germinated seedlings.

In this work, we used the seed-specific promoter of *JcSDP1* for regulated expression of the *JcSDP1*-related RNAi construct. The *JcSDP1* promoter was chosen because its activity peaks in the early stage of developing seed, and gradually returns to basal levels in the late stage (Figure 4E). Moreover, the *JcSDP1* promoter is responsive to sugars, especially sucrose and fructose (Figure 4D). Based on these findings, we used the *JcSDP1* promoter to express *JcSDP1*-related RNAi sequence so as to establish a feed-back inhibition system. For instance, if the gene silencing is not strong enough to knock down *JcSDP1* expression, the residual JcSDP1 would degrade TAGs into FFA, which can be converted into sucrose, trehalose, and proteins. Once sucrose is elevated to a certain critical level in the endosperm, it can activate the *JcSDP1* promoter, which in turn enhances suppression of *JcSDP1* gene expression. The gene silencing strategy we showed here has several advantages over the use of a CaMV35S promoter: 1) using the cognate promoter of a target gene can offer more specific regulation of the RNAi*-*dependent gene silencing against the target gene itself; 2) the end product of the target gene can be used for its own feed-back suppression; and 3) there is reduced ectopic expression of transgene.

### *SDP1*-deficient transgenic Jatropha plants may be advantageous for the process of biodiesel production

Biodiesel is commonly produced from crude oil by alkaline or acid treatment processes, which are known as transesterification [34,35]. Owing to its shorter reaction time and reduced energy consumption, the alkaline-treatment process is the preferred method for transesterification. For this process, crude oil should contain a very low levels of FFA and moisture because a high level of FFA and water can transform transesterification into saponification, leading to easy depletion of catalysts [36,37]. For crude oil containing a high level of FFA and water, acid treatment is more suitable, but it entails a longer reaction time with a greater requirement for alcohol. This dilemma has prompted studies to improve transesterification processes by chemical methods [38,39]. We anticipate that the deficiency of seed-specific *SDP1* in Jatropha plant may provide a solution to this dilemma. FFA levels of mature seeds are mainly determined by SDP1 during the late maturation stage [15,29]. Here, we showed that dry, mature seeds of *JcSDP1-*RNAi transgenic lines contain higher TAG levels and lower FFA levels compared with control (CK; 35S:*GFP*) Jatropha plants (see Additional file 5). Therefore, crude oils derived from *SDP1*-deficient transgenic Jatropha plants would be expected to be a better substrate compared with those of control (CK; 35S:*GFP*) plants for alkaline transesterification in biodiesel production. However, to confirm the positive effect of *SDP1* deficiency, a detailed analysis of alkaline-treated transesterification with crude oils from *JcSDP1-*RNAi transgenic plants should be performed in future studies.

## Conclusion

We have shown that *SDP1* deficiency enhances seed lipid accumulation in the model plant Arabidopsis. Based on this result, we used RNAi technology and the native *JcSDP1* promoter to generate transgenic Jatropha plants with reduced endogenous *SDP1* expression. We showed that *SDP1*-deficient transgenic Jatropha plants accumulate around 30% higher seed total lipid than control (CK; 35S:*GFP*) plants. Our transgenic technology has therefore resulted in an enhanced agronomic trait for this biodiesel crop.

## Methods

### Plant materials and growth condition

Arabidopsis Col-0 was grown on soil with sand. Seeds of the homozygous *sdp1-5* mutant (*Salk_076697*, Arabidopsis Biological Resource Center (ABRC), Ohio State University, Columbus, OH, USA) were stratified at 4°C for 3 days on soil and germinated, then seedlings were grown on in a growth chamber under 16 hour light/8 hour dark cycles, at 23°C?±?3°C under white light (100 to 150 μE/m/s photosynthetically active radiation). Rosette leaves were harvested from 3 to 4-week-old plants, and other organs such as inflorescence stems, cauline leaves, flowers, siliques, and developing seeds were harvested from 6-week-old plants. We considered seeds from siliques at 3 to 5 days after pollination) to be at the early stage of seed maturation in Arabidopsis. We collected leaves and developing fruits (S1: 1 week after fertilization (WAF) S2: 2 to 3 WAF, S3: 4 to 6 WAF, and S4: 7 to 8 WAF) from Jatropha plants grown in a greenhouse in Singapore.

### RNA isolation and quantitative real-time PCR

Total RNA was isolated from plant samples using TRIzol reagent (Invitrogen, Carlsbad, CA USA) in accordance with the manufacturer’s instructions. cDNA was synthesized with 1 μg total RNA using MMLV Superscript II (Promega, Madison, WI, USA) after DNase I treatment (Roche Applied Science, Mannheim, Germany). Quantitative real-time PCR assay was performed on an ABI 7900 sequence Detection System (Applied Biosystems, Foster City, CA, USA). We used power SYBR Green PCR Master Mix (Applied Biosystems), using the manufacturer’s reagent protocol, but reducing the volume to 10 μl per reaction. As controls, we used the species-specific *tubulin* and *actin* primer sets for *J. curcas* and *A. thaliana*, respectively. Fold change values of the target gene transcripts were subsequently normalized by dividing the ^∆^Ct values by the ^∆^Ct values of each control gene transcript. All real-time PCR experiments were performed in triplicate using different biological samples. Sequences of primers used in PCR procedures are listed in Additional file 7.

### Isolation of *JcSDP1* full-length cDNA and its promoter

We found a partial *SDP1* sequence in our *J. curcas* expressed sequence tag database. Using this partial sequence, we designed 5′ or 3′ cDNA RACE primers and performed RACE experiments using BD SMART™ RACE cDNA Amplification Kit (Clontech, Mountain View, CA, USA). We used 1 μg of total RNA derived from developing seeds (S2 to S3) for generation of 5′ or 3′ RACE pools. The 5′ and 3′ cDNA ends were obtained by touchdown PCR with the Advantage 2 PCR Enzyme System (Clontech), following the manufacturer’s instructions. To amplify the products specifically, the primary RACE products were diluted and mixed with a nested gene-specific primer (GSP2) and nested universal primer mixture (AP2). The PCR products were gel-purified and cloned into pDrive Cloning vector (Qiagen, Düsseldorf, Germany) to obtain full-length *JcSDP1* cDNA (2577-bp). A Universal GenomeWalker Kit (Clontech) was used to isolate the promoter fragment of the *JcSDP1* gene. Genomic DNA was digested with *Dra*I, *Eco*RV, *Pvu*II, *Ssp*I, and *Stu*I endonucleases, and five libraries of adaptor-ligated genomic fragments were constructed. These genomic DNA libraries were used as templates for the PCR for promoter isolation. For each round of genome walking, the primary PCR products were amplified by a gene-specific primer (GSP1) and the outer adaptor primer (AP1). For the second PCR mix, the primary products were diluted and used as templates with a nested gene-specific primer (GSP2) and the nested adaptor primer (AP2). The secondary PCR products were then separated in agarose gels, and the relevant DNA fragment purified with QIAEXII Gel Extraction Kit (Qiagen), cloned into pDrive Cloning vector, and sequenced. Potential *cis* elements in the promoter region were analyzed using computational analytical methods available on two public websites, PlantCare (http://bioinformatics.psb.ugent.be/webtools/plantcare/html/) and PLACE (http://www.dna.affrc.go.jp/PLACE/).

### Construction of the *ProJcSDP1:GUS* fusion gene and analysis of promoter

We used the pKGWFS7 destination vector including *GFP* or *GUS* reporter gene for promoter analysis. The 5′-flanking region of *JcSDP1* was amplified using two specific primers, *JcSDP1*-PF1T and *JcSDP1*-PR1. This promoter was 873 bp in length, including 149 bp of 5′-UTR. The amplified PCR product was inserted into a TOPO donor vector using the pENTR^TM^/D-TOPO Cloning Kit (Invitrogen), and then inserted into the destination vector, pKGWFS7, using the Gateway LR Clonase™ II enzyme mix (Invitrogen). For plant transformation, the constructs were introduced by electroporation into *Agrobacterium tumefaciens strain* AGL1. Constructs were transformed into WT (Col-0) or *sdp1-5* mutant (*Salk_076697*) background through the floral dip method described by Clough and Bent [40].

For transient assay of *JcSDP1* promoter expression, 10 μg of *ProJcSDP1:GUS* in pKGWFS7 plasmid DNA was coated with 1 μm diameter gold particles (2.5 mg gold particles, 200 μl of 2.5 M CaCl_2_ and 100 μl of 0.1 M spermidine). After being incubated on ice for 30 minutes, the pellet of DNA/gold particle was washed twice with 70% ethanol and resuspended in 100% ethanol. Jatropha fruits and leaves were centered in a petri dish containing MS agar medium [41]. Fruits and leaves were bombarded at 1,350 psi with a biolistic helium gun device (PDS-1000/He, Bio-rad, Hercules, CA, USA). After incubation for 2 days at 25°C, the bombarded tissues were analyzed by histochemical assays as described previously by Jefferson *et al*. [42]. Tissues were incubated in GUS staining buffer (0.1 M Sodium phosphate pH 7.0, 1 mM 5-bromo-4-chloro-3-indolyl-D-glucuronide (Sigma-Aldrich, St Louis, MO, USA), 0.5 mM potassium ferrocyanide, 0.5 mM potassium ferricyanide, 10 mM Na_2_EDTA, and 0.1% Triton X-100) for 20 hours at 37°C. Stained tissues were rinsed with 70% to 80% ethanol until pigments had been cleared. Selected organs transgenic Arabidopsis lines expressing *ProJcSDP1:GUS* were analyzed for GUS expression. To investigate sugar-responsive expression of *JcSDP1* promoter, we used 14-day-old seedlings of T2 lines, seedlings were incubated for 24 hours in an MS media with 1% or 3% of a sugar source: sucrose, fructose, and glucose. Mannitol was used as a control for osmotic stress.

### Complementation of the *JcSDP1*/*sdp1-5* mutation in Arabidopsis

Full-length *JcSDP1* cDNA was cloned into a vector harboring a tandem CaMV35S promoter and a Nos polyA addition sequence from pCAMBIA 1300, and transformed into Arabidopsis *sdp1-5* mutant (*Salk_076697*). T1 transformants were selected on 40 μg/ml hygromycin, and T2 seedlings were assayed for sugar responsiveness on MS agar plates with or without sucrose.

### Construction of inducible marker-free *ProJcSDP1:JcSDP1-*RNAi vector and transformation of Jatropha plants

We used the pCCreloxP (pCCLB3) inducible marker-free vector system as described by Qiu *et al*. [43]. The transfer DNA (T-DNA) of pCCreloxP vector harbors a *loxP* fragment that consists of *CRE-int*, *HPT* and *XVE* genes (Figure 5A). To silence *JcSDP1* expression using RNAi, we selected a 373-bp fragment that includes 290 bp of the 3′ coding region of *JcSDP1* cDNA with a stop codon and 83 bp of the 3′ UTR region. The 373-bp *JcSDP1* fragment was amplified with two primer sets (*JcSDP1*-RNAiF-*Xho*I and *JcSDP1*-RNAiR2-*Hind*III or *JcSDP1*-RNAiF-*BamH*I and *JcSDP1*-RNAiR2-*Pst*I, used for the sense orientation or anti-sense orientation, respectively), and the fragments were then cloned into pBluescript-SK intron vector (pBS-SK*i*) which has an intron (156 bp) to generate p*JcSDP1-*RNAi as previously described [44]. *ProJcSDP1* was amplified with two primers, *JcSDP1*-PF1-*Apa*I and *JcSDP1*-PR1-*Xho*I, and then restricted with *Apa*I/*Xho*I. To insert the *ProJcSDP1* (*JcSDP1* promoter) fragment into the p*JcSDP1-*RNAi construct, we used *Apa*I and *Xho*I restriction enzyme sites in the construct. A 226-bp fragment containing the CaMV35S polyA addition sequence (T35S) was amplified with T35S-F-*Xba*I and T35S-R-*Pm*l-*Sac*II primers. The amplified T35S fragment was cleaved with *Xba*I/*Sac*II restriction enzymes, and then cloned into *ProJcSDP1-*RNAi. Finally, the *ProJcSDP1*:*JcSDP1-*RNAi:*T*_*35S*_ fragment from the *ProJcSDP1-*RNAi construct was inserted into the pCCreloxP marker-free vector at the *Apa*I and *Pml*I sites. All constructs were introduced into *A. tumefaciens* strain AGL1 using electroporation, and then transformed into Jatropha using cotyledon explants [45].

### Fatty acid analysis in Arabidopsis and Jatropha

We used GC to analyze the FA profile of *sdp1-5* as described by Li *et al*. [46]. For this, 100 dried seeds of each line were weighed, and samples were transmethylated at 85°C for 2 hours in a reaction buffer (1 ml of 3 M HCl-methanol, 25 μl of butylhydroxytoluene solution, and 300 μl of toluene (all Sigma-Aldrich)]. As an internal standard, 50 μg of pentadecanoic acid (C15:0; Sigma-Aldrich) was added to each sample. After the samples had been cooled down to room temperature, 1.5 ml of 0.9% NaCl (w/v) was added to the mix, and the FA methyl esters (FAMEs) were extracted two times with 1 ml of hexane. Extracts were evaporated under nitrogen and then dissolved in 100 μl of hexane. The final extracts were analyzed with GC using a flame ionization detector (FID) on Agilent 6890 (Agilent, Santa Clara, CA, USA) employing helium as the carrier gas. Total FAs were estimated by comparing the total FAME peak area (pA*sec) to that of the C15:0.

To analyze total lipid content in Jatropha transgenic lines, we used three changes of hexane 3 to extract all lipid components in dried endosperm [7], which includes mainly TAG, and sterols, FFA, DAG, and monoacylglycerol (MAG) components. To profile FA composition, about 10 mg of total lipid were transmethylated at 70°C for 20 minutes in a reaction buffer [1 ml of 3 N methanolic-HCl, 400 μl of 2,2, dimethoxypropane (Sigma-Aldrich) and 50 μg of pentadecanoic acid (C15:0)]. After being cooled down to room temperature, the FAMEs were extracted by twice 1 ml of water and 1 ml of hexane extraction. Extracts were evaporated under nitrogen and then dissolved in 500 μl of hexane. The final samples were analyzed in a GCMS-QP2010 (Shimadzu, Japan). SD was calculated based on several different plants.

### Analysis of lipids by TLC and quantification by GC/MS

Seed lipids were extracted with hexane three times [7,46]. After determination of total lipid amount, 300 μg of neutral lipid were fractionated by TLC on silica gel plates in a running solvent mixture (hexane: ethyl acetic acid: acetic acid; 90:10:1, respectively, by volume). Triolein (T7140; Sigma-Aldrich) and oleic acid (75090; Sigma-Aldrich) were used as a standard of TAG and of FFA, respectively. TLC plates were exposed to iodine (I_2_) vapor for visualization. The separated neutral lipid species, including TAG and FFA, were recovered from the plates using hexane, and quantified by GC/MS after conversion to their corresponding methyl esters by the methanolic-HCl method as described by Li *et al*. [46]. The absolute amount was calculated using C15:0 as an internal standard and by comparing the relative peak areas.

### Protein and carbohydrate analysis

Protein content in Jatropha transgenic lines was analyzed as described by Focks and Benning [47], using 50 mg of dried endosperm. Protein amounts were measured by the Lowry DC protein assay (Bio-Rad) using γ-globulin as a standard. To analyze carbohydrate content, 50 mg of dried endosperm were homogenized in 200 μl of assay buffer and centrifuged at full speed. The extracted supernatant was used for carbohydrate quantification using a Total Carbohydrate Assay Kit (Sigma-Aldrich). D-glucose was used as a standard for calibration.

### Genomic DNA isolation and Southern blotting

Total genomic DNA was isolated from leaves from transgenic or control (CK; 35S:*GFP*) plants grown in a greenhouse, using cetyltrimethylammonium bromide (Sigma-Aldrich) [48]. Genomic DNA was digested with restriction enzymes and separated in 0.8% agarose gels. The gels were processed and blotted onto Hybond-N^+^ membranes (Roche Applied Science) following standard procedures [49]. Probes were prepared with PCR DIG Labelling Mix using specific primer sets for the *hygromycin phosphotransferase* (*HPT*) gene and the *JcSDP1* gene. Hybridization was performed using PCR DIG detection kit following the supplier’s instructions (Roche Applied Science).

### TEM and SEM

TEM was performed with mature dried seeds of WT (Col-0) and *sdp1-5* mutant. Seeds were embedded in resin and sectioned on an ultramicrotome (Leica Ultracut UCT; Leica, Wetzlar, Germany) set at 70 nm thickness. Sectioned samples were placed onto 300 mesh copper grids. Sections were examined, and pictures were taken with a TEM (JEM-1230; JEOL, Tokyo, Japan) at 120 kV. For SEM analysis, dried seeds were mounted directly and examined under a JSM-6360LV SEM (JEOL) with an acceleration voltage of 20 kV.

### Seed weight and size measurement

Mature seeds were harvested from WT (Col-0) and *sdp1-5* mutant grown under the same conditions. A sample size of 100 seeds per WT (Col-0) or *sdp1-5* mutant was used to obtain an average seed weight with at least five biological replications. Values (n?=?5) are given as mean?±?SD. A DM5000B microscope (Leica) and ImageJ analysis software were used to measure seed sizes. Values (n?=?10) are given as mean?±?SD.

## Abbreviations

DAP: Days after pollination; CoA: coenzyme A; FAME: Fatty acid methyl ester; FFA: Free fatty acid; GC: Gas chromatography; GC/MS: Gas chromatography/mass spectrometry; GFP: Green fluorescent protein; GUS: β-Glucuronidase; PTLs: Patatin-like TAG lipases; qPCR: Quantitative PCR; RNAi: RNA interference; SEM: Scanning electron microscopy; S1P: Site-1 protease; SDP1: Sugar-dependent1; TAGs: Triacylglycerol; T-DNA: transfer DNA; TEM: Transmission electron microscopy; TLC: Thin layer chromatography; WAF: Weeks after fertilization.

## Competing interests

The results described in this work were the subject of a patent application filed by the Temasek Life Sciences Laboratory.

## Authors’ contributions

MJK, SWY, and JLY designed the experiments, and MJK, JLY, HZM, and SPV performed the experiments. All authors reviewed, discussed, and interpreted the results. MJK and NHC wrote the manuscript which was reviewed and approved by all authors.

## Supplementary Material

Additional file 1: Figure S1 Schematic diagram of *Arabidopsis thaliana* (At) *SDP1* gene structure and insertion position of T-DNA in *sdp1-5* allele. (A) The T-DNA is inserted in the first exon of *SDP1* resulting in a null mutation. (B) Isolation of the homozygous *sdp1-5* mutant by RT PCR. (C) Expression levels of *AtSDP1* in early developing seeds (3 to 5 DAP) of WT (Col-0) and *sdp1-5. Actin1* (At2g37620) expression levels were used as an internal control. *P?<?0.05, **P?<?0.01, ***P?<?0.001 versus WT (Col-0) for five biological replicates. SD, standard deviation (n?=?5). Col-0, Columbia-0; DAP, days after pollination. Click here for file

Additional file 2: Table S1 Seed weight and size of wild type (WT) (Columbia-0; Col-0) and *sdp1-5.*^a^Seed weight determination using 100 mature seeds. Values (n?=?5) are given with as mean?±?SD. ^b^The length and width of seed were measured using mature dried seeds. Values are given (n?=?10) as mean?±?SD. *P?<?0.05, **P?<?0.01 or ***P?<?0.001 versus WT (Col-0) seed. Click here for file

Additional file 3: Figure S2 Relative expression levels of fatty acid synthesis-related genes in early developing seeds of wild type (WT) (Columbia-0; Col-0) and *sdp1-5.* The cDNA library was synthesized from total mRNA derived from seeds (3 to 5 DAP). Values are given as mean?±?SD (n?=?3). *ACP1* (acyl carrier protein; At3g05020), *FAD2* (oleate desaturase; At3g12120), *FAD3* (linoleate desaturase; At2g29980), *FAE1* (fatty acid elongase; At4g34520), *Cy-PK* (cytosol pyruvate kinase; At5g52920), *Ch-PK* (chloroplast pyruvate kinase: At3g22960), *KASI* (ketoacyl-ACP Synthase I; At5g46290), *KASIII* (ketoacyl-ACP synthase III; At1g62640), *BCCP2* (biotin carboxyl carrier protein; At5g15530), and *SUS2* (sucrose synthase 2; At5g49190). DAP, days after pollination. Click here for file

Additional file 4: Table S2 Protein and carbohydrate contents in endosperm of control (CK; 35S:*GFP*) and *JcSDP1-*RNAi transgenic plants. Values are the mean?±?SE of measurements on endosperms from individual seed (n?=?4) of *JcSDP1-*RNAi #158 T1 transgenic and control (CK; 35S:*GFP*) plants grown in a greenhouse. RNAi, RNA interference. Click here for file

Additional file 5: Figure S3 Analysis of lipid composition of mature dried endosperm of *JcSDP1-*RNAi transgenic line #158 by thin layer chromatography (TLC) and gas chromatography/mass spectrometry (GC/MS). (A) Total lipids were extracted from mature endosperm of control (CK; 35S:*GFP*) plants and *JcSDP1-*RNAi transgenic line #158, and 300 μg of total neutral lipids were separated by TLC on silica gel plate and stained with iodine (I_2_). (B) Relative amounts of FFA and TAG determined by GC/MS. The absolute amount was calculated using the C15:0 as an internal control by comparing their peak areas. (C) Relative fatty acid level of FFAs in endosperm of control (CK; 35S:*GFP*) plant and *JcSDP1-*RNAi transgenic line #158 by GC/MS. (D) Relative fatty acid level of TAGs in endosperm of control (CK; 35S:*GFP*) plant and *JcSDP1-*RNAi transgenic line #158 by GC/MS. FFA, free fatty acid; DAG, diacylglycerol; RNAi, RNA interference; SE, sterol ester; TAG, triacylglycerol. Click here for file

Additional file 6: Figure S4 Southern blot analysis of T0 and T1 transgenic plants expressing *JcSDP1-*RNAi*.* (A) Total genomic DNA were digested with *Xho*I restriction enzyme and hybridized with *HPT* probe. (B) Total lipid content (% w/w) in individual transgenic line carrying the *JcSDP1-*RNAi construct. RNAi, RNA interference. Click here for file

Additional file 7: Table S3 Sequences of primers used in the experiments. Click here for file
