# Supplementary figures and images for: Gene silencing of Sugar-dependent 1 (JcSDP1), encoding a patatin-domain triacylglycerol lipase, enhances seed oil accumulation in Jatropha curcas
Source: Biotechnol Biofuels. 2014 Mar 8;7:36. doi: 10.1186/1754-6834-7-36 (PMC4016141; doi:10.1186/1754-6834-7-36)

**Figure S1**

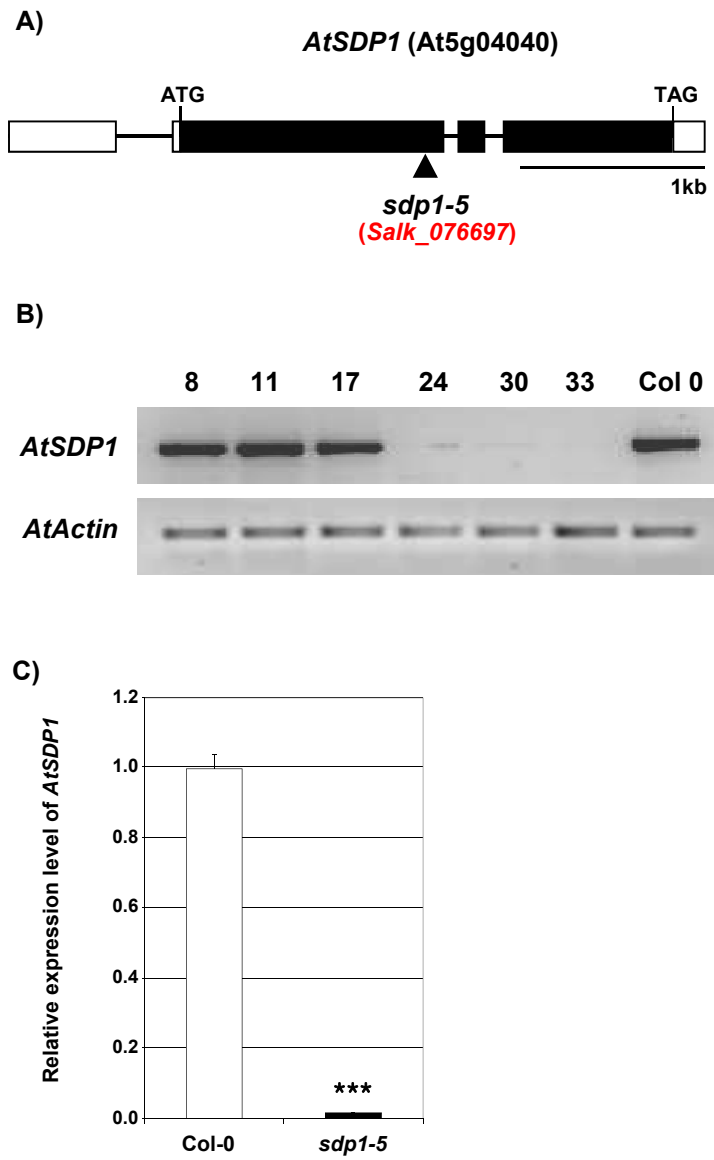

Supplement: Additional file 1: Figure S1 — Schematic diagram of Arabidopsis thaliana (At) SDP1 gene structure and insertion position of T-DNA in sdp1-5 allele. (A) The T-DNA is inserted in the first exon of SDP1 resulting in a null mutation. (B) Isolation of the homozygous sdp1-5 mutant by RT PCR. (C) Expression levels of AtSDP1 in early developing seeds (3 to 5 DAP) of WT (Col-0) and sdp1-5. Actin1 (At2g37620) expression levels were used as an internal control. *P?<?0.05, **P?<?0.01, ***P?<?0.001 versus WT (Col-0) for five biological replicates. SD, standard deviation (n?=?5). Col-0, Columbia-0; DAP, days after pollination. [file 1754-6834-7-36-S1.pdf]

Figure S2

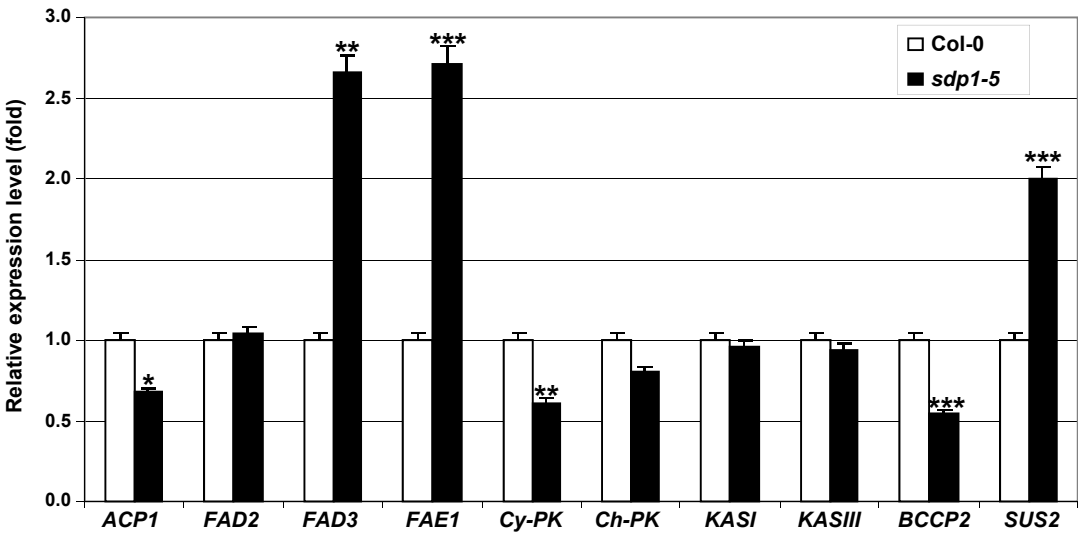

Supplement: Additional file 3: Figure S2 — Relative expression levels of fatty acid synthesis-related genes in early developing seeds of wild type (WT) (Columbia-0; Col-0) and sdp1-5. The cDNA library was synthesized from total mRNA derived from seeds (3 to 5 DAP). Values are given as mean?±?SD (n?=?3). ACP1 (acyl carrier protein; At3g05020), FAD2 (oleate desaturase; At3g12120), FAD3 (linoleate desaturase; At2g29980), FAE1 (fatty acid elongase; At4g34520), Cy-PK (cytosol pyruvate kinase; At5g52920), Ch-PK (chloroplast pyruvate kinase: At3g22960), KASI (ketoacyl-ACP Synthase I; At5g46290), KASIII (ketoacyl-ACP synthase III; At1g62640), BCCP2 (biotin carboxyl carrier protein; At5g15530), and SUS2 (sucrose synthase 2; At5g49190). DAP, days after pollination. [file 1754-6834-7-36-S3.pdf]

Figure S3

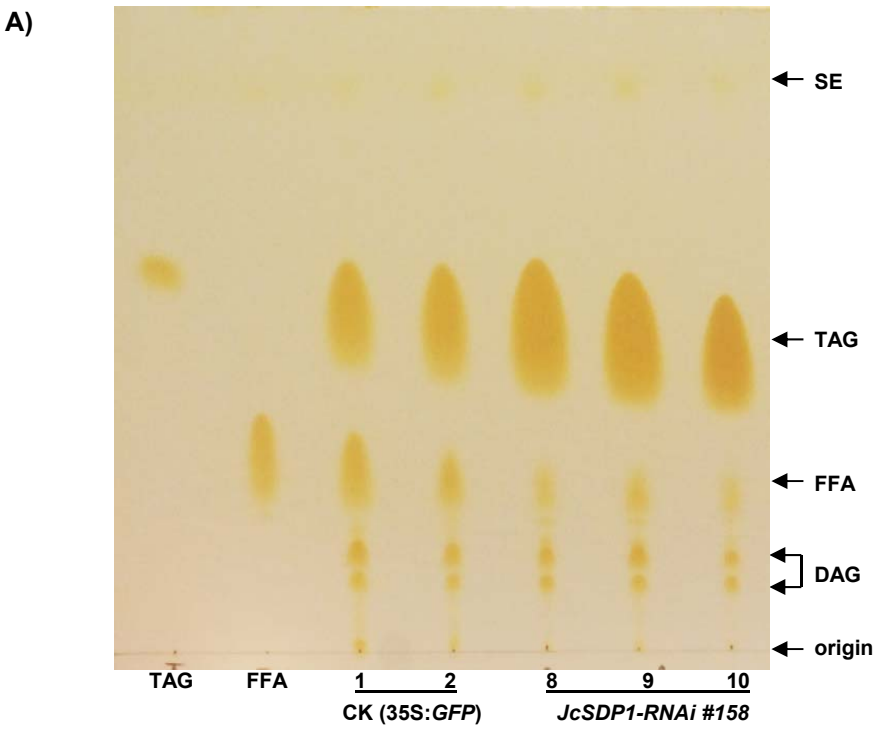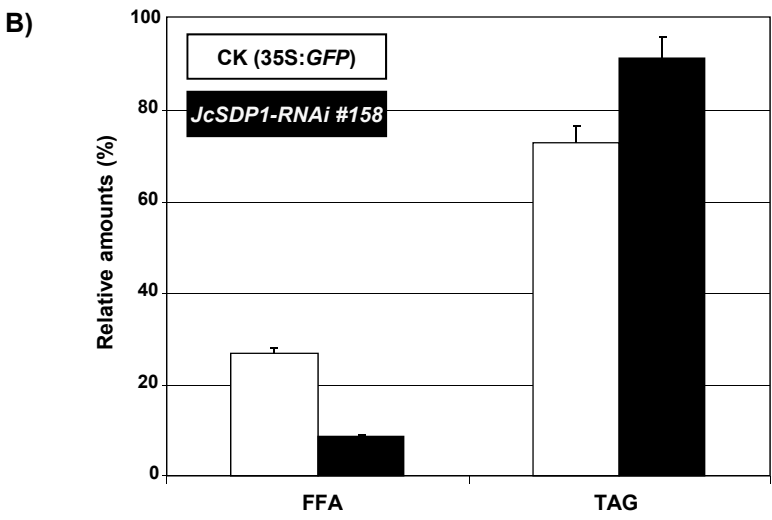

Figure S3

C)

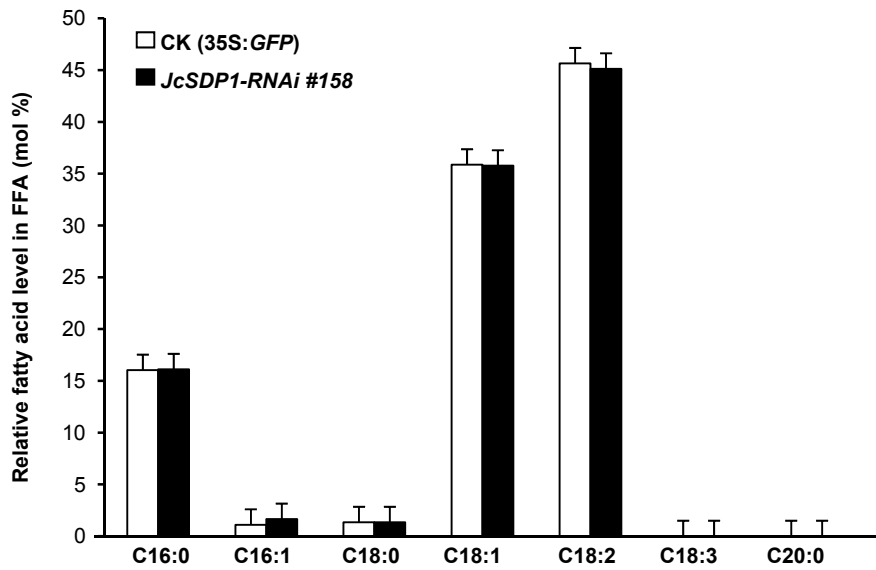

D)

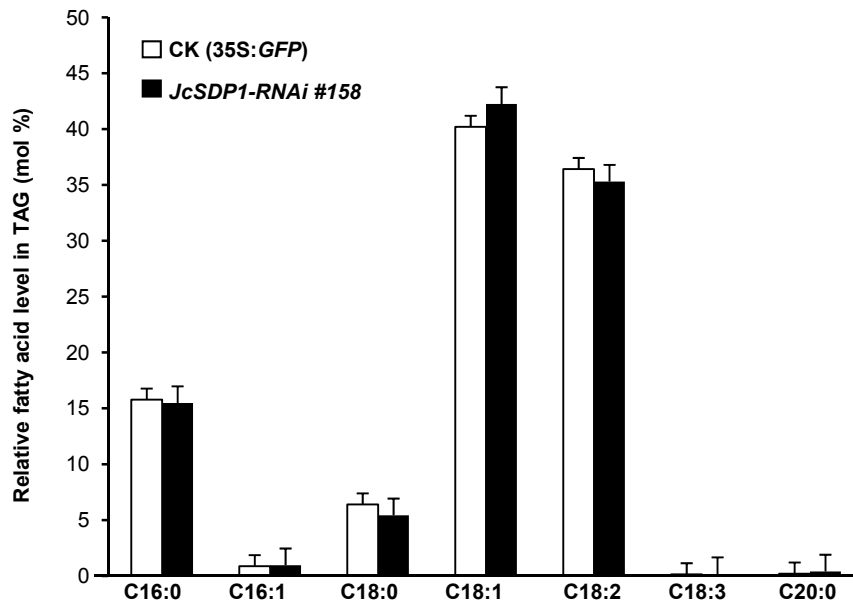

Supplement: Additional file 5: Figure S3 — Analysis of lipid composition of mature dried endosperm of JcSDP1-RNAi transgenic line #158 by thin layer chromatography (TLC) and gas chromatography/mass spectrometry (GC/MS). (A) Total lipids were extracted from mature endosperm of control (CK; 35S:GFP) plants and JcSDP1-RNAi transgenic line #158, and 300 μg of total neutral lipids were separated by TLC on silica gel plate and stained with iodine (I2). (B) Relative amounts of FFA and TAG determined by GC/MS. The absolute amount was calculated using the C15:0 as an internal control by comparing their peak areas. (C) Relative fatty acid level of FFAs in endosperm of control (CK; 35S:GFP) plant and JcSDP1-RNAi transgenic line #158 by GC/MS. (D) Relative fatty acid level of TAGs in endosperm of control (CK; 35S:GFP) plant and JcSDP1-RNAi transgenic line #158 by GC/MS. FFA, free fatty acid; DAG, diacylglycerol; RNAi, RNA interference; SE, sterol ester; TAG, triacylglycerol. [file 1754-6834-7-36-S5.pdf]

Figure S4

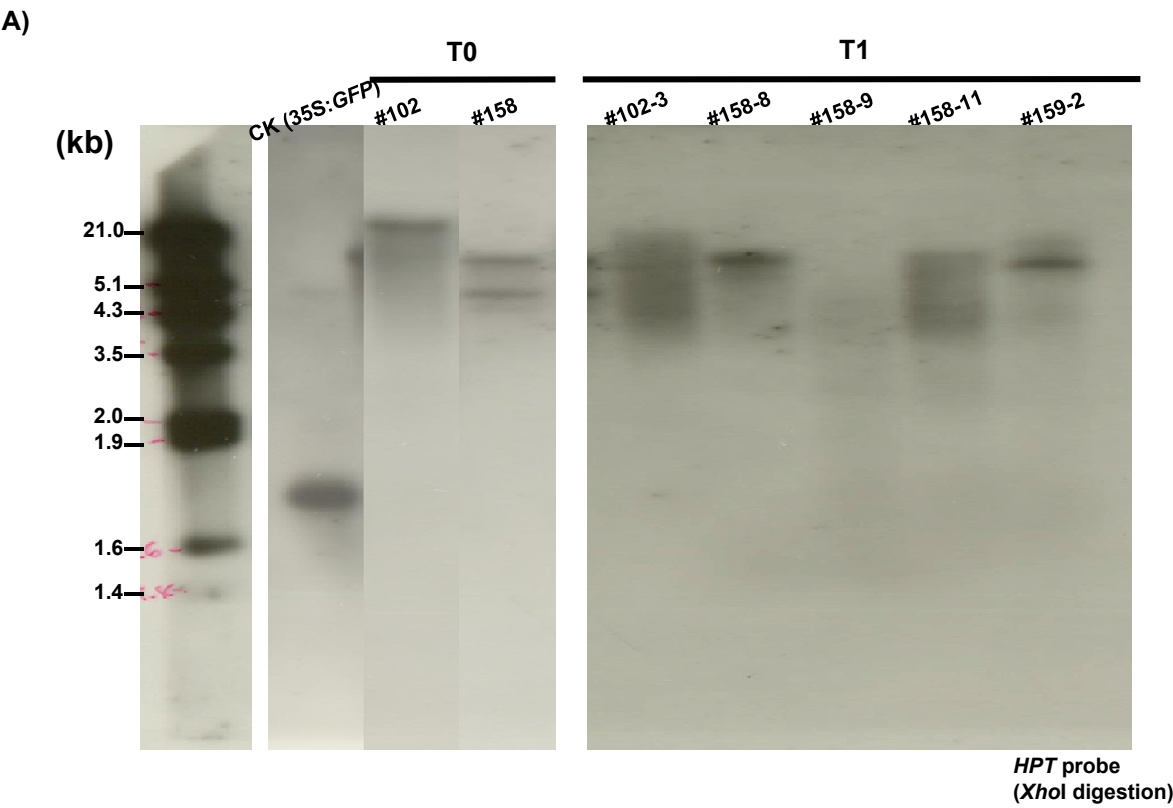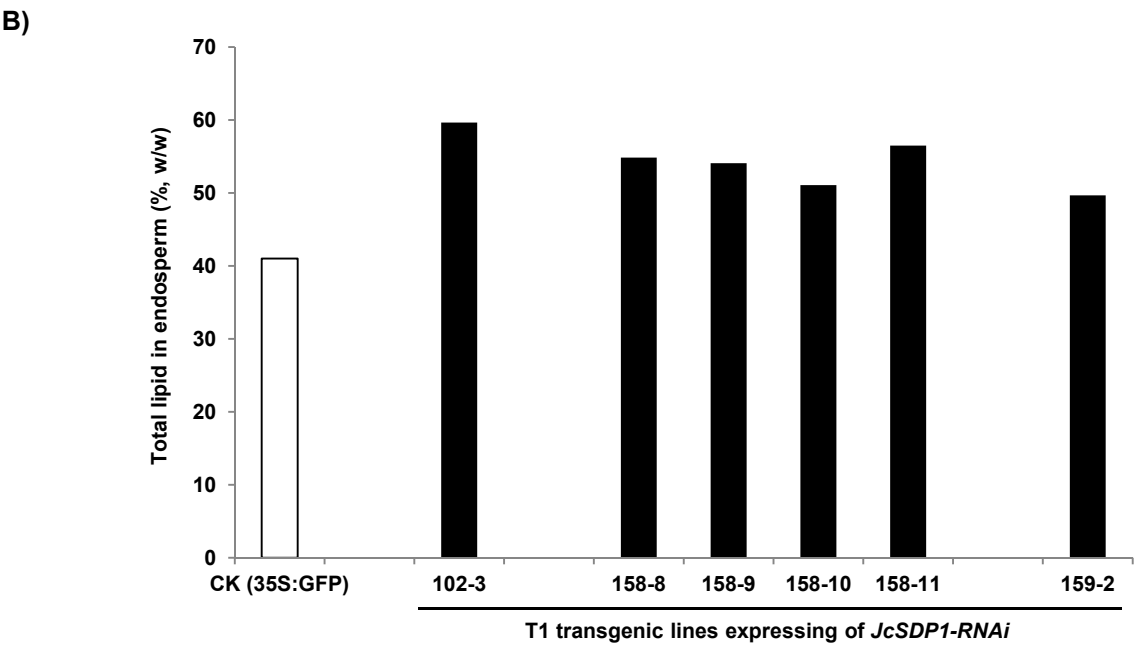

Supplement: Additional file 6: Figure S4 — Southern blot analysis of T0 and T1 transgenic plants expressing JcSDP1-RNAi. (A) Total genomic DNA were digested with XhoI restriction enzyme and hybridized with HPT probe. (B) Total lipid content (% w/w) in individual transgenic line carrying the JcSDP1-RNAi construct. RNAi, RNA interference. [file 1754-6834-7-36-S6.pdf]
