# Supplementary material for: Gene silencing of Sugar-dependent 1 (JcSDP1), encoding a patatin-domain triacylglycerol lipase, enhances seed oil accumulation in Jatropha curcas
Source: Biotechnol Biofuels. 2014 Mar 8;7:36. doi: 10.1186/1754-6834-7-36 (PMC4016141; doi:10.1186/1754-6834-7-36)
Supplement: Additional file 2: Table S1 — Seed weight and size of wild type (WT) (Columbia-0; Col-0) and sdp1-5.aSeed weight determination using 100 mature seeds. Values (n?=?5) are given with as mean?±?SD. bThe length and width of seed were measured using mature dried seeds. Values are given (n?=?10) as mean?±?SD. *P?<?0.05, **P?<?0.01 or ***P?<?0.001 versus WT (Col-0) seed. [file 1754-6834-7-36-S2.pdf]

Table S1

Seed weight and size of WT (Col-0) and *sdp1-5*

Name	Weight of seed (μg/seed) [a]		Length of seed (μm/seed) [b]		Width of seed (μm/seed) [b]	
Col-0	19.00	±1.10	481.12	±3.36	276.70	±10.58
sdp1-5	21.00	±0.57 **[c]	522.86	±7.56***	294.29	±15.12 *

[a]Seed weight determination using 100 mature seeds. Average values are given with SD (n=5).
[b]The length and width of seed were measured using mature dried seeds. Average values are given with SD (n=10).
[c]*P<0.05, **P<0.01 or ***P<0.001 versus WT (Col -0) seed.
