# Supplementary material for: Gene silencing of Sugar-dependent 1 (JcSDP1), encoding a patatin-domain triacylglycerol lipase, enhances seed oil accumulation in Jatropha curcas
Source: Biotechnol Biofuels. 2014 Mar 8;7:36. doi: 10.1186/1754-6834-7-36 (PMC4016141; doi:10.1186/1754-6834-7-36)
Supplement: Additional file 4: Table S2 — Protein and carbohydrate contents in endosperm of control (CK; 35S:GFP) and JcSDP1-RNAi transgenic plants. Values are the mean?±?SE of measurements on endosperms from individual seed (n?=?4) of JcSDP1-RNAi #158 T1 transgenic and control (CK; 35S:GFP) plants grown in a greenhouse. RNAi, RNA interference. [file 1754-6834-7-36-S4.pdf]

Table S2

Protein and Carbohydrate content in endosperm of control (CK; 35S:*GFP*) and *JcSDP1-RNAi* transgenic plants

Genotype	Total lipid content (%, w/w)	Protein content (%, w/w)	Carbohydrate content (%, w/w)
CK (35S:*GFP*)	41.41 ± 2.19	14.93 ± 0.16	13.89± 0.12
JcSDP1-RNAi #158	54.17 ± 2.13	13.99 ± 0.19	13.92± 0.13

Values are the mean ± SE of measurements on endosperms from individual seed (n=4) of *JcSDP1-RNAi* #158 T1 transgenic and CK (35S:*GFP*) plant s grown in a greenhouse.
