# Supplementary material for: Gene silencing of Sugar-dependent 1 (JcSDP1), encoding a patatin-domain triacylglycerol lipase, enhances seed oil accumulation in Jatropha curcas
Source: Biotechnol Biofuels. 2014 Mar 8;7:36. doi: 10.1186/1754-6834-7-36 (PMC4016141; doi:10.1186/1754-6834-7-36)
Supplement: Additional file 7: Table S3 — Sequences of primers used in the experiments. [file 1754-6834-7-36-S7.pdf]

Table S3

Sequences of primers used in the experiments

**Promoter analysis**		
	JcSDP1-PF1T	**CACC**tacatccagtagattgcacgtcacacacta
	JcSDP1-PR1	ctctaaaagtttcgtgttttggatgatttgct
**Gene construction**		
	JcSDP1-CF-Sal	ttggtcgacatggatataagtaatgaggccaatgt
	JcSDP1-CR-Nhe	attgctagcaccatccacagaactttgatcctgtc
	JcSDP1-RNAiF-Xho	atactcgagtccataatgggattgtgttgaacgtcgt
	JcSDP1-RNAiR2-H3	tcgaagcttgctagtggcacttgatattaaggtgat
	JcSDP1-RNAiF-Bm	ataggatcctccataatgggattgtgttgaacgtcgt
	JcSDP1-RNAiR2-Pst	tctttctgcagctagtggcacttgatattaaggt
	JcSDP1-PF1-ApaI	tatgggccctacatccagtagattgcacgtcacaca
	JcSDP1-PR1-Xho	atgctcgagctctaaaagtttcgtgttttggatgattt
	T35S-F-Xba	acttctagacggccatgctagagtccgcaa
	T35S-R-Pml-SacII	ttaccgcgg**cacgtg**aggtcactggatttt
**MF genotyping**		
	HygF	aaaaagcctgaactcaccgcgacgtct
	HygR	tacttctacacagccatcggtcca
	MF-P1	ctgaattgtcgaggtcgaagatc
	JcSDP1-R16	agcagccaaatgggtctgtcc
	T35S-R	cttctcattatcggtggtgaacat
**qRT-PCR**		
At2g37620	AtActin F	ctctgtctggattggagggtc
	AtActin R	gcttgagaaatggtcggaaa
At4g25140	AtOleo1-F	tgccgctataaccgttttctcttgga
	AtOleo1-R	atgttccccaccagtatgttgctgtcc
At5g40420	AtOleo2-F	atgcggttggctacgcaggacaa
	AtOleo2-R	tcatgcagccgtcgtcctcccct
At5g51210	AtOle3-F	tggaatagccgccattaccgcctt
	AtOle3-R	agaaacttgttggtgttggactccaatg
At5g04040	AtSDP1-QF	atgccatcgattgggaacca
	AtSDP1-QR	caccggttcttgtaaaccggaat
	JcTubulin F	gaggctggatctggcaaacacgtt
	JcTubulin R	tgtgtaatgacctctagcaaaatta
	JcSDP1-QF	cataatgggattgtgttgaacgtcgt
	JcSDP1-QR	gtcatcatcgtcatccccagcata
**Fatty acid related genes in Arabidopsis**		
At3g12120	AtFAD2-F	cctcagcctctctcttacttggctt
	AtFAD2-R	ccaggagaagtaagggacgag
At2g29980	AtFAD3-F	ctacttgttggtccataatgttcgtca
	AtFAD3-R	cgtagataactccattccttgcct
At4g34520	AtFAE1-F	gtcttaaccaactttttcaacctctgtt
	AtFAE1-R	ccaaaccgaaaacagtgaaagcaaagagt
At3g05020	AtACP1-F	aatctatccttcaacctccgccgt
	AtACP1-R	agagaatctgctccaaggtcagct
At5g15530	AtBCCP2-F	ggatctcctttccctccgatgttt
	AtBCCP2-R	ataaattcagagagctcggcgggt
At3g22960	AtCh-PK-F	tggcatcactggtctcccgatgt
	AtCh-PK-R	tcatacctcttgatttcagtaacgaga
At5g52920	AtCy-PK-F	agtcactatcgtccttccggcacaa
	AtCy-PK-R	cgctctgtacgattgctatttcctct
At5g49190	*AtSUS2-F*	tcaagtcagttcactgcagatctaat
	AtSUS2-R	caactcggtaaagaccaggcatagtgaa
At5g46290	AtKAS1-F	tcgcaaaacacacatcacacac
	AtKAS1-R	gtgattgacgatttgatggtaag
At1g62640	AtKASIII-F	tggtcttcctgcttctgcc
	AtKASIII-R	ccgcttctcactgcctcat
